# *Lactobacillus delbrueckii* CRL 581 Differentially Modulates TLR3-Triggered Antiviral Innate Immune Response in Intestinal Epithelial Cells and Macrophages

**DOI:** 10.3390/microorganisms9122449

**Published:** 2021-11-27

**Authors:** Mariano Elean, Leonardo Albarracin, Kohtaro Fukuyama, Binghui Zhou, Mikado Tomokiyo, Shugo Kitahara, Shota Araki, Yoshihito Suda, Lucila Saavedra, Julio Villena, Elvira M. Hebert, Haruki Kitazawa

**Affiliations:** 1 Reference Centre for Lactobacilli (CERELA-CONICET), Tucuman 4000, Argentina; melean@cerela.org.ar (M.E.); lalbarracin@herrera.unt.edu.ar (L.A.); lucila@cerela.org.ar (L.S.); 2 Laboratory of Animal Food Function, Food and Feed Immunology Group, Graduate School of Agricultural Science, Tohoku University, Sendai 980-8572, Japan; kotaro.fukuyama.p8@dc.tohoku.ac.jp (K.F.); zhou.binghui.s5@dc.tohoku.ac.jp (B.Z.); mikado.tomokiyo.t4@dc.tohoku.ac.jp (M.T.); shugo.kitahara.p6@dc.tohoku.ac.jp (S.K.); shota.araki.t4@dc.tohoku.ac.jp (S.A.); 3 International Education and Research Center for Food Agricultural Immunology (CFAI), Livestock Immunology Unit, Graduate School of Agricultural Science, Tohoku University, Sendai 980-8572, Japan; 4 Department of Food, Agriculture and Environment, Miyagi University, Sendai 980-8572, Japan; suda@myu.ac.jp

**Keywords:** TLR3, *Lactobacillus delbrueckii* subsp. *lactis* CRL 581, antiviral response, intestinal epithelial cells, macrophages, immunobiotics

## Abstract

*Lactobacillus delbrueckii* subsp. *lactis* CRL 581 beneficially modulates the intestinal antiviral innate immune response triggered by the Toll-like receptor 3 (TLR3) agonist poly(I:C) in vivo. This study aimed to characterize further the immunomodulatory properties of the technologically relevant starter culture *L. delbrueckii* subsp. *lactis* CRL 581 by evaluating its interaction with intestinal epithelial cells and macrophages in the context of innate immune responses triggered by TLR3. Our results showed that the CRL 581 strain was able to adhere to porcine intestinal epithelial (PIE) cells and mucins. The CRL 581 strain also augmented the expression of antiviral factors (IFN-α, IFN-β, Mx1, OAS1, and OAS2) and reduced inflammatory cytokines in PIE cells triggered by TLR3 stimulation. In addition, the influence of *L. delbrueckii* subsp. *lactis* CRL 581 on the response of murine RAW macrophages to the activation of TLR3 was evaluated. The CRL 581 strain was capable of enhancing the expression of IFN-α, IFN-β, IFN-γ, Mx1, OAS1, TNF-α, and IL-1β. Of note, the CRL 581 strain also augmented the expression of IL-10 in macrophages. The results of this study show that the high proteolytic strain *L. delbrueckii* spp. *lactis* CRL 581 was able to beneficially modulate the intestinal innate antiviral immune response by regulating the response of both epithelial cells and macrophages relative to TLR3 activation.

## 1. Introduction

Diarrhea and gastroenteritis caused by rotavirus is one of the most important causes of hospitalization and mortality in children. Although the mortality and morbidity associated with rotavirus infection in infants was diminished in recent years by vaccine applications, such intestinal infection is still a persistent problem globally. It is estimated that at least 200,000 young children die of diarrhea annually due to the severe infections produced by this virus [1,2,3]. Intestinal epithelial cells (IECs) located in the villi of the small intestine are the main target of rotavirus, and its replication induces villous blunting and malabsorption [4,5]. The virus is recognized by IECs by using pattern-recognition receptors (PRRs) including retinoic acid-inducible gene-I (RIG-I), melanoma differentiation-associated gene-5 (MDA-5) and Toll-like receptor (TLR)-3. The activation of PRRs by viral molecules such as double stranded RNA (dsRNA) stimulates signaling cascades in IECs that induce the biosynthesis of type I interferons (IFNs), antiviral factors and inflammatory cytokines and chemokines that coordinate the innate antiviral immune response mediated by neighboring IECs and immune cells such as resident macrophages [6,7]. In addition to the signals provided by infected IECs, intestinal macrophages can directly recognize rotavirus [8]. The activation of PRRs signaling pathways in macrophages also induces the production of type I and type II IFNs as well as inflammatory cytokines in response to rotavirus infection. Macrophages are also activated in phagocyte infected cells and present antigens. Then, macrophages have an important role in suppressing viral replication and inducing effective adaptive immune responses.

Research has demonstrated that TLR3 has a key role in the outcome of rotavirus infection. Studies in humans showed that individuals older than 5 years are often asymptomatic while young infants frequently develop gastroenteritis after rotavirus infection [9]. Of note, the same report showed that the expression levels of TLR3 in the intestinal epithelium of children below 5 years were significantly lower than in individuals 5–20 years of age. In line with these observations in humans, it was observed that adult and young mice differ in their resistance to rotavirus infection. While adult mice remained asymptomatic and shed low numbers of rotavirus particles, infants and neonatal mice are highly susceptible to infection [9]. The expression of TLR3 in the intestinal epithelium correlated with the different susceptibility of adult versus young mice with respect to the viral infection. In fact, TLR3 expression was lower in young mice than in adult animals. Furthermore, it was reported that adult mice deficient in TLR3 are highly susceptible to rotavirus infection compared with wild-type animals [9]. Of note, it was shown that rotavirus or its purified dsRNA are capable of inducing severe mucosal damage in the gut via the inflammation triggered by the activation of TLR3 conducting mucosal erosion, villous atrophy, and gut wall attenuation [10]. Then, for the elimination of rotavirus with minimal damage to the intestinal mucosa, it is of utmost importance to maintain an appropriate balance between the protection induced by the TLR3-mediated innate immune response and the inflammatory damage.

The scientific community actively searches for effective alternatives for the prevention or treatment of intestinal infections produced by enteric viruses such as rotavirus. In this regard, the use of beneficial immunomodulatory probiotic microorganisms referred to as “immunobiotics” [11,12] has been proposed to improve the outcome of viral diarrhea [13,14,15]. Furthermore, it was proposed that the selection of immunobiotic strains that have both remarkable immunomodulatory and biotechnological properties could be helpful for potentiating the development of new types of functional foods that could be used to reduce the incidence and severity of infections caused by intestinal viruses in high-risk populations such as infants. We hypothesized that use of proteolytic starter strains that have intrinsic immunomodulatory properties could allow the development of milk-based antiviral foods using a single bacterium, diminishing the cost of their manufacture. Thus, taking into consideration that successful milk fermentation is achieved by lactic acid bacteria (LAB) with highly proteolytic activity [16,17], we recently evaluated several proteolytic *Lactobacillus delbrueckii* strains and selected the ones with the capacity to modulate innate antiviral immune responses in mice [18]. Among the strains evaluated, *L. delbrueckii* subsp. *lactis* CRL 581 stood out because of its immunomodulatory properties. The CRL 581 strain augmented phagocytic activities of peritoneal macrophages and increased the levels of intestinal IgA and IFN-γ after its oral administration to mice. Furthermore, we demonstrated that *L. delbrueckii* subsp. *lactis* CRL 581 beneficially influenced the intestinal antiviral innate immune response since it was able to significantly augment the intestinal levels of IFN-γ and IFN-β in mice and protect against intestinal inflammatory damage after the challenge with the TLR3 agonist poly(I:C) [18]. Furthermore, this strain is capable of fermenting milk, releasing bioactive peptides with demonstrated antihypertensive properties both in vitro and in vivo [19,20]. This makes the *L. delbrueckii* subsp. *lactis* CRL 581 strain a relevant functional starter culture.

The interaction of the immunobiotic lactobacilli with epithelial and immune cells in the intestinal mucosa has been widely studied. Several reports have described that the beneficial effects of immunobiotics in the intestinal immune system depends largely on the molecular interactions that occur between lactobacilli with IECs and antigen-presenting cells (APCs) such as macrophages, which are the first cells of the host to come into contact with microorganisms that reach the intestinal mucosa [12,15,21]. Then, this study aimed to further characterize the immunomodulatory properties of the technologically relevant starter culture *L. delbrueckii* subsp. *lactis* CRL 581 by evaluating its interaction with IECs and macrophages in the context of the innate immune response triggered by the activation of TLR3.

## 2. Materials and Methods

### 2.1. Lactobacilli Growth Conditions and Case in Hydrolysates Preparation

*L. delbrueckii* subsp. *lactis* CRL 581 belongs to the CERELA-CONICET Culture Collection (Tucuman, Argentina). *Ligilactobacillus salivarius* FFIG58 and *L. salivarius* FFIG79 strains belong to the Food and Feed Immunology Group Collection (Sendai, Japan). The lactobacilli strains were grown twice in MRS (de Man, Rogosa and Sharpe) broth (Biokar Diagnostics) in capped test tubes at 37 °C for 16 h. In order to eliminate carryover of nutrients, lactobacilli were harvested by centrifugation (9000× *g*, 10 min) and washed twice in sterile 0.85% (*w*/*v*) saline solution. Bacteria were then resuspended in this solution to the original volume for experiments [18]. The β-casein hydrolysate produced by the CRL 581 strain (CRL 581 hydrolysate) was obtained, as described before [18]. Briefly, bacterial cells were grown in a chemically defined medium at the exponential growth phase (OD_600_ = 0.8) and then harvested by centrifugation (8000× *g*, 10 min at 4 °C). The buffer Tris-HCl (100 mM, pH 7.5) was used to wash twice the bacterial cells. The pellets were resuspended in buffer Tris-HCl (100 mM, pH 7.5) and concentrated 50 times. Cell fractions from lactobacilli containing the proteinase were mixed with 5 mg/mL of β-casein (Sigma-Aldrich) dissolved in 0.1 M of Na-phosphate buffer (pH 7.0) at 1:1 volume ratio. The mixtures were incubated at 40 °C for 4 h. The samples were centrifuged, and supernatants were stored at −20 °C until use.

### 2.2. Porcine Intestinal Epithelial Cells and TLR3 Activation

The porcine intestinal epithelial (PIE) cell line was developed from intestinal epithelia of an unsuckled neonatal pig at Tohoku University, as described previously [22]. Dulbecco’s Modified Eagle’s Medium (DMEM) supplemented with 10% fetal calf serum (FCS), streptomycin (100 mg/mL), and penicillin (100 U/mL) was used for PIE cell cultures. The cells (3.0 × 10^4^ per well) were grown in 12 well type I collagen (bovine dermis) coated plates at 37 °C in a humidified atmosphere of 5% CO_2_. After 3 days of culture, 1 mL of DMEM containing *L. delbrueckii* subsp. *lactis* CRL 581 (5 × 10^8^ cells/mL) or its β-casein hydrolysate (CRL 581 hydrolysate, 300 μg) was added to PIE cell monolayers for 48 h. Then, PIE cells were washed to eliminate stimulants and challenged with poly(I:C) (60 μg/mL) for 12 or 24 h. The expressions of interferon (IFN)-α, IFN-β, interferon regulatory factor 3 (IRF3), zinc finger protein A20 (A20), IFN-induced GTP-binding protein Mx1 (Mx1), ribonuclease L (RNAseL), 2′-5′-oligoadenylate synthetase 1 (OAS1), OAS2, regenerating islet-derived protein 3γ (REG3γ), S100 calcium-binding protein A8 (S100A8), interleukin 6 (IL-6), IL-8, C-C motif chemokine ligand 2 (CCL2 or monocyte chemoattractant protein 1, MCP-1), CCL4 (or macrophage inflammatory protein-1β, MIP-1β), and C-X-C motif chemokine ligand 5 (CXCL5 or epithelial-derived neutrophil-activating peptide 78, ENA-78) were determined by RT-qPCR as described below.

### 2.3. RT-qPCR in PIE Cells

The expression of immune factors in PIE cells was evaluated by RT-qPCR, as described previously [22,23]. Briefly, the TRIzol reagent (Invitrogen) was used to extract total RNA, and its purity and quantity were analyzed by a Nano drop spectrophotometer ND-1000 UV-Vis (NanoDrop Technologies, Wilmington, DE, USA). cDNA was synthesized with RNA (500 ng) using a Thermal cycler (BIO-RAD, Hercules, CA, USA) with the Quantitect reverse transcription (RT) kit (Qiagen, Tokyo, Japan) and by considering the instructions of the manufacturer. A 7300 real-time PCR system (Applied Biosystems, Warrington, UK) with platinum SYBR green (Invitrogen) was used to perform the qPCR. The primers for the immune factors were described previously [22,23]. For the PCR reaction, 2.5 μL of cDNA was made into a mixture with 7.5 μL of master mix that included the following: forward and reverse primers (1 pmol/μL), RT enzyme, and SYBR green. The reaction cycles were performed as follows: 50 °C for 5 min; 95 °C for 5 min; 40 cycles at 95 °C for 15 s, 60 °C for 30 s, and finally 72 °C for 30 s. The β-actin gene was used as a housekeeping gene because of its high stability across various porcine tissues [24]. The expression of β-actin was used to normalize cDNA levels for differences in total cDNA levels in the samples. A relative index was calculated after normalization with β-actin, and the results were expressed as normalized fold expression based on cell controls set at 1.0.

### 2.4. Adhesion to PIE Cells

The adhesion of *L. delbrueckii* subsp. *lactis* CRL 581 to PIE cells was performed using fluorescent bacteria and the microplate method, as described previously [25]. In these experiments, *L. salivarius* FFIG58 and *L. salivarius* FFIG79 strains [25] were used for comparisons. Lactobacilli were cultured, washed with PBS three times (6000 rpm, 10 min), and resuspended in 1 mL PBS. Then, 1 mM of carboxyfluorescein diacetate (CFDA) was added for the fluorescent reaction at 37 °C for 1 h. Lactobacilli were washed with PBS three times (6000 rpm, 10 min) to remove CFDA from the microbial surface. Fluorescent bacteria were counted by a hemocytometer. PIE cells were seeded at 5000 cells/well in Type I collagen-coated 96 well cell culture plates (Nippi Incorporated, Tokyo, Japan) for 3 days. Fluorescent CRL 581, FFIG58 and FFIG79 bacteria were added to different culture plates of PIE cells at 100 MOI and cultured for 48 h. After incubation, non-adherent lactobacilli were eliminated by washing with PBS. NaOH 0.1 N was used for lysis, and fluorescence was evaluated by a 2030 Multilabel Reader (Perkin Elmer, Fukuoka, Japan).

### 2.5. Adhesion to Porcine Mucins

For the evaluation of the adhesion of *L. delbrueckii* subsp. *lactis* CRL 581 to porcine mucins, biacore experiments were performed using a Biacore 1000 (GE Healthcare Bio-Sciences K.K., Shield, UK) at 25 Cin ab HBS-EP buffer and purified porcine mucins, as described previously [25]. The highly adherent *L. salivarius* FFIG79 and the low adherent *L. salivarius* FFIG58 strains [25] were used for comparisons. An amine coupling reaction was used for the immobilization of purified porcine mucins on a CM5 sensor chip (GE Healthcare Bio-Sciences K.K.) by considering the instructions of the manufacturer. The reaction between *N*-hydroxysuccinimide-esters and radicals of primary amino groups present in mucins molecules was used to immobilize mucins (10 mg/mL in 10 mM sodium acetate buffer, pH 4.0). An HBS-EP buffer was used to equilibrate the sensor chip. Adhesion using the Biacore 1000 is based on the principle of surface plasmon resonance. After washing and lyophilization, lactobacilli were suspended in an HBS-EP buffer (3 mg/mL).

The bacterial suspension was injected at a flow rate of 3 μL/min for 5 min, and the HBS-EP buffer was used to remove unbound analytes from the sensor chip. The regeneration was performed by eluting 1 M guanidine hydrochloride (GHCl) solution at a flow rate of 3 μL/min for 2 min. The resonance units (RU) were measured for 200 s after the cessation of sample addition. A response of 1 RU represents 1 pg/mm^2^ protein adhering with an increased concentration of analyte bound to the sensor chip’s surface.

### 2.6. Murine Macrophages Cultures

The mouse macrophage cell line RAW 264.7 was cultured in DMEM supplemented with 10% FCS, 100 IU/mL penicillin, and 100 mg/mL streptomycin and maintained at 37 °C in a 5% CO_2_ humidified incubator. One mL of DMEM containing *L. delbrueckii* subsp. *lactis* CRL 581 (5 × 10^8^ cells/mL) or the CRL 581 hydrolysate (300 μg) was added to macrophages monolayers. Macrophage stimulation was performed from 12 h, and cells were then washed to eliminate the stimulants and challenged with poly(I:C) (50 μg/mL). The expressions of IFN-α, IFN-β, IFN-γ, Mx1, OAS1, tumor necrosis factor (TNF)-α, IL-1α, IL-1β, IL-10, and IL-27 were determined by RT-qPCR 12 h after poly(I:C) challenge.

### 2.7. RT-qPCR in Macrophages

Total RNA was isolated from macrophages using TRIzol reagent (Invitrogen). All cDNAs were synthesized using a Quantitect Reverse-Transcription (RT) Kit (Qiagen, Tokyo, Japan) according to the instruction of the manufacturer. A 7300 Real-Time PCR System (Applied Biosystems, Warrington, UK) and the Platinum SYBR Green qPCR SuperMix (Invitrogen, Carlsbad, CA, USA) were used to carried our qPCR. The primers used in this study were described previously [26]. The PCR cycling conditions were 2 min at 50 °C, followed by 2 min at 95 °C, and then 40 cycles of 15 s at 95 °C, 30 s at 60 °C, and 30 s at 72 °C. The reaction mixtures contained 5 μL of sample cDNA and 15 μL of the master mix, which included sense and antisense primers. The expression of the housekeeping gene was used to normalize cDNA levels for differences in total cDNA levels in the samples. A relative index was calculated after normalization with β-actin, and the results were expressed as normalized fold expression based on cell controls set as 1.0.

### 2.8. Statistical Analysis

Statistical analyses were performed using GLM and REG procedures available in the SAS computer program (SAS, 1994). Comparisons between mean values were carried out using one-way analysis of variance and Fisher’s least-significant-difference (LSD) test. For these analyses, *p* values of < 0.05 were considered significant.

## 3. Results

### 3.1. Adhesion Properties of L. delbrueckii subsp. lactis CRL 581

**Figure 1:**
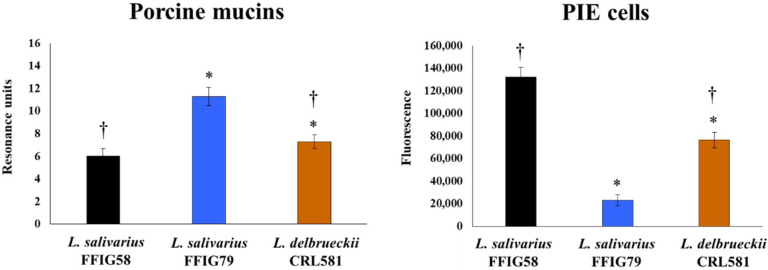
Adhesion of *Lactobacillus delbrueckii* subsp. *lactis* CRL 581 to porcine intestinal epithelial (PIE) cells and porcine mucins. The porcine *Ligilactobacillus salivarius* strains FFIG58 and FFIG79, which are highly adherent to PIE cells and porcine mucins, respectively, were used for comparisons. The results represent data from three independent experiments. Asterisks indicate significant differences when compared to the FFIG58 strain (* *p* < 0.05). Symbols indicate significant differences when compared to the FFIG79 strain (^†^ *p* < 0.05).

An important property of probiotic lactobacilli is their ability to colonize the gastrointestinal tract. In this manner, probiotic bacteria interact with IECs and immune cells of the intestinal mucosa by exerting their beneficial effects. Lactobacilli’s colonization ability is related to their capacity to bind to mucus and IECs. We evaluated the capacity of *L. delbrueckii* subsp. *lactis* CRL 581 in adhering to porcine mucins and PIE cells. *L. salivarius* isolated from the intestinal tract of pigs posessing different adhesion properties [25] was used for comparisons. *L. delbrueckii* subsp. *lactis* CRL 581 showed the ability to adhere to porcine mucins (Figure 1), with intermediate results between the high adherent FFIG79 and low adherent FFIG58 strains. 

In addition, *L. delbrueckii* subsp. *lactis* CRL 581 was capable of adhering to PIE cells in contrast to the FFIG79 strain, which had no ability to adhere to cells as shown by the fluorescence units that were not different from control cells (Figure 1). Of note, the adhesion capacity of the CRL 581 strain was modest when compared to *L. salivarius* FFIG58.

### 3.2. Immunomodulatory Properties of L. delbrueckii subsp. lactis CRL 581 in PIE Cells

**Figure 2:**
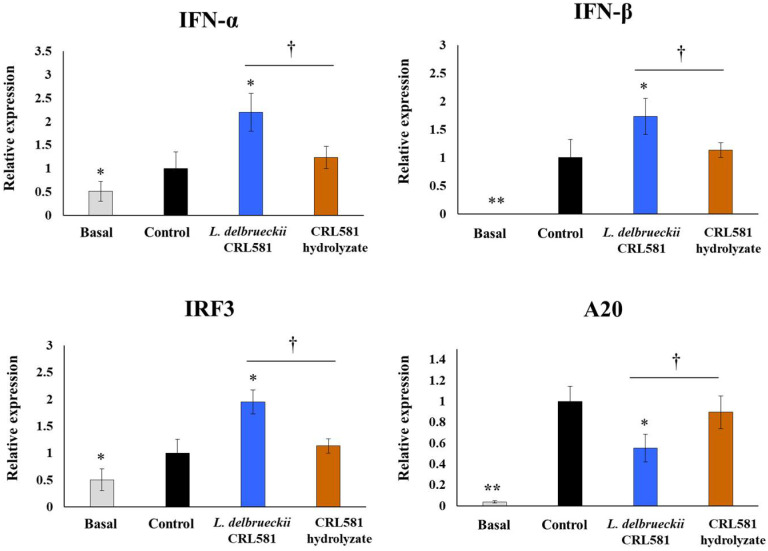
Modulation of the innate antiviral immune response triggered by Toll-like receptor 3 (TLR3) activation in porcine intestinal epithelial (PIE) cells by *Lactobacillus delbrueckii* subsp. *lactis* CRL 581. PIE cells were stimulated with *L. delbrueckii* subsp. *lactis* CRL 581 or its β-casein hydrolysate (CRL 581 hydrolysate) and challenged with poly(I:C) to induce the activation of TLR3. PIE cells with no treatment (basal) or challenged only with poly(I:C) (control) were used as controls. The expressions of interferon (IFN)-α, IFN-β, the interferon regulatory factor 3 (IRF3), and zinc finger protein A20 (A20) were analyzed by qPCR after 12 h of TLR3 activation. The results represent data from three independent experiments. Asterisks indicate significant differences when compared to the control group (* *p* < 0.05, ** *p* < 0.01). Symbols indicate significant differences between the indicated groups (^†^ *p* < 0.05).

We next analyzed the effect of *L. delbrueckii* subsp. *lactis* CRL 581 on the innate antiviral immune response induced by TLR3 activation in PIE cells. For this purpose, PIE cells were stimulated with the CRL 581 strain or its β-casein hydrolysate (CRL 581 hydrolysate) and then challenged with a TLR3 agonist. Twelve hours after poly(I:C) stimulation, the expression of IFN-α and IFN-β was evaluated (Figure 2).

The challenge with poly(I:C) significantly increased the expression of both type I IFNs in PIE cells. Cells treated with *L. delbrueckii* subsp. *lactis* CRL 581 had significantly higher levels of type I IFNs (IFN-α/β) than control cells, while the CRL 581 hydrolysate treatment induced no modifications in the expression of type I IFNs when compared to the control group (Figure 2). The TLR3 agonist also induced a significant increase in the expression of IRF3 in all the experimental groups. However, PIE cells treated with the CRL 581 strain had levels of IRF3 that were significantly higher than controls. In addition, activation of TLR3 by poly(I:C) augmented the expression levels of the regulatory protein A20. Of note, CRL 581-treated PIE cells had levels of A20 that were lower than controls. The expressions of IRF3 and A20 in PIE cells stimulated with the CRL 581 hydrolysate were not statistically different from controls (Figure 2).

**Figure 3:**
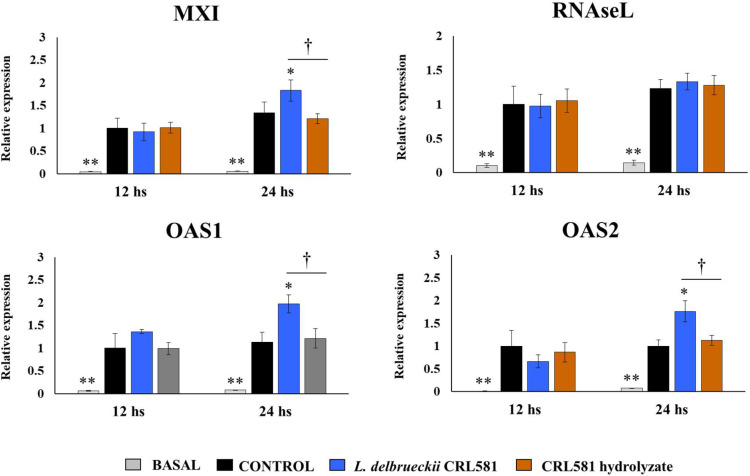
Modulation of the innate antiviral immune response triggered by Toll-like receptor 3 (TLR3) activation in porcine intestinal epithelial (PIE) cells by *Lactobacillus delbrueckii* subsp. *lactis* CRL 581. PIE cells were stimulated with *L. delbrueckii* subsp. *lactis* CRL 581 or its β-casein hydrolysate (CRL 581 hydrolysate) and challenged with poly(I:C) to induce the activation of TLR3. PIE cells with no treatment (basal) or challenged only with poly(I:C) (control) were used as controls. The expressions of interferon-induced GTP-binding protein Mx1 (Mx1), ribonuclease L (RNAseL), 2′-5′-oligoadenylate synthetase 1 (OAS1), and OAS2 were analyzed by qPCR after 12 and 24 h of TLR3 activation. The results represent data from three independent experiments. Asterisks indicate significant differences when compared to the control group (* *p* < 0.05, ** *p* < 0.01). Symbols indicate significant differences between the indicated groups (^†^ *p* < 0.05).

Considering that *L. delbrueckii* subsp. *lactis* CRL 581 augmented the expression of type I IFNs, we also analyzed the changes in the expression of antiviral factors that are regulated by IFNs including Mx1, RNAseL, OAS1, and OAS2 (Figure 3).

Poly(I:C) stimulation induced a significant increase in the expression of the four antiviral factors in all experimental groups and with no significant differences between them at hour 12 post-stimulation. However, when the expressions of antiviral factors were evaluated at hour 24 after poly(I:C) challenge, PIE cells treated with *L. delbrueckii* subsp. *lactis* CRL 581 had significantly higher levels of Mx1, OAS1, and OAS2 than controls (Figure 3). The expressions of Mx1, RNAseL, OAS1, and OAS2 in PIE cells stimulated with the CRL 581 hydrolysate were not different from controls at hour 24 after poly(I:C) challenge (Figure 3).

**Figure 4:**
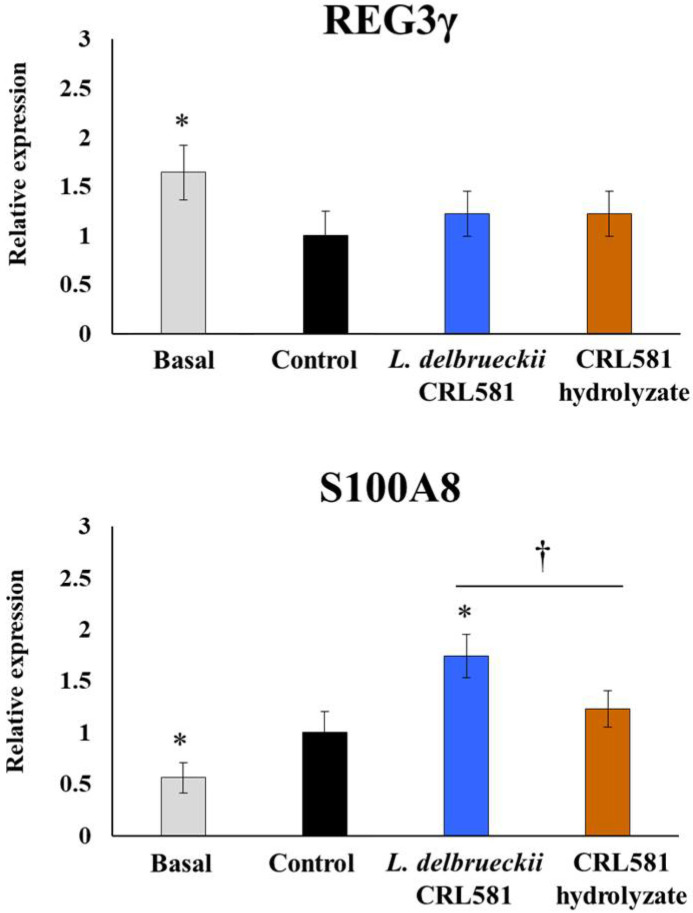
Modulation of the innate antiviral immune response triggered by Toll-like receptor 3 (TLR3) activation in porcine intestinal epithelial (PIE) cells by *Lactobacillus delbrueckii* subsp. *lactis* CRL 581. PIE cells were stimulated with *L. delbrueckii* subsp. *lactis* CRL 581 or its β-casein hydrolysate (CRL 581 hydrolysate) and challenged with poly(I:C) to induce the activation of TLR3. PIE cells with no treatment (basal) or challenged only with poly(I:C) (control) were used as controls. The expressions of regenerating islet-derived protein 3γ (REG3γ) and S100 calcium-binding protein A8 (S100A8) were analyzed by qPCR after 12 h of TLR3 activation. The results represent data from three independent experiments. Asterisks indicate significant differences when compared to the control group (* *p* < 0.05). Symbols indicate significant differences between the indicated groups (^†^ *p* < 0.05).

The expressions of the antimicrobial peptides regenerating islet-derived protein 3γ (REG3γ) and S100 calcium-binding protein A8 (S100A8) were also evaluated in PIE cells. The activation of TLR3 significantly reduced the expression of REG3γ in all the experimental groups and with no differences between them (Figure 4).

In contrast, poly(I:C) stimulation augmented the levels of S100A8 in PIE cells. Of note, PIE cells treated with *L. delbrueckii* subsp. *lactis* CRL 581 had significantly higher levels of S100A8, while cells treated with the CRL 581 hydrolysate were not different from controls (Figure 4). The expressions of Mx1, RNAseL, OAS1, and OAS2 in PIE cells stimulated with the CRL 581 hydrolysate were not different from controls at hour 24 after poly(I:C) challenge (Figure 3).

**Figure 5:**
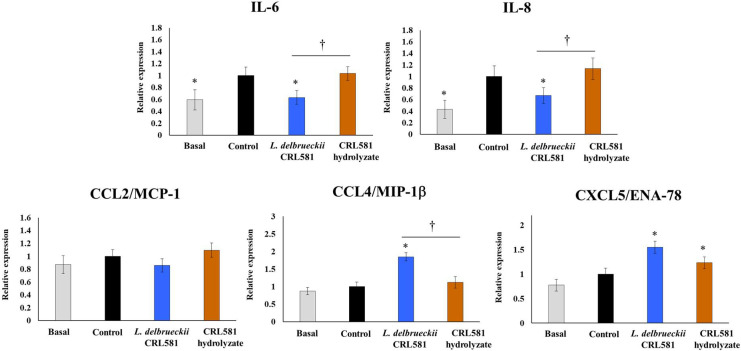
Modulation of the innate antiviral immune response triggered by Toll-like receptor 3 (TLR3) activation in porcine intestinal epithelial (PIE) cells by *Lactobacillus delbrueckii* subsp. *lactis* CRL 581. PIE cells were stimulated with *L. delbrueckii* subsp. *lactis* CRL 581 or its β-casein hydrolysate (CRL 581 hydrolysate) and challenged with poly(I:C) to induce the activation of TLR3. PIE cells with no treatment (basal) or challenged only with poly(I:C) (control) were used as controls. The expressions of interleukin 6 (IL-6), IL-8, C-C motif chemokine ligand 2 (CCL2 or monocyte chemoattractant protein 1, MCP-1), CCL4 (or macrophage inflammatory protein-1β, MIP-1β), and C-X-C motif chemokine ligand 5 (CXCL5 or epithelial-derived neutrophil-activating peptide 78, ENA-78) were analyzed by qPCR after 12 and 24 h of TLR3 activation. The results represent data from three independent experiments. Asterisks indicate significant differences when compared to the control group (* *p* < 0.05). Symbols indicate significant differences between the indicated groups (^†^ *p* < 0.05).

We also characterized the changes in the expression of inflammatory cytokines and chemokines in PIE cells after the activation of TLR3. As shown in Figure 5, poly(I:C) challenge increased the expression of IL-6, IL-8, CCL4/MIP-1, and CXCL5/ENA-78, while the expression of CCL2/MCP-1 was not different form the basal group.

PIE cells treated with *L. delbrueckii* subsp. *lactis* CRL 581 had significantly lower levels of IL-6 and IL-8 than the controls (Figure 5). Furthermore, the levels of these two cytokines had values that were comparable to those observed in the basal group. In addition, CRL 581-stimulated PIE cells showed a higher expression of CCL4/MIP-1β and CXCL5/ENA-78 than controls after the challenge with poly(I:C). The expressions of the inflammatory cytokines and chemokines in PIE cells stimulated with the CRL 581 hydrolysate were not different from controls after poly(I:C) challenge with the exception of CXCL5/ENA-78 that was increased in the hydrolysate-treated cells (Figure 5).

### 3.3. Immunomodulatory Properties of L. delbrueckii subsp. lactis CRL 581 in Macrophages

**Figure 6:**
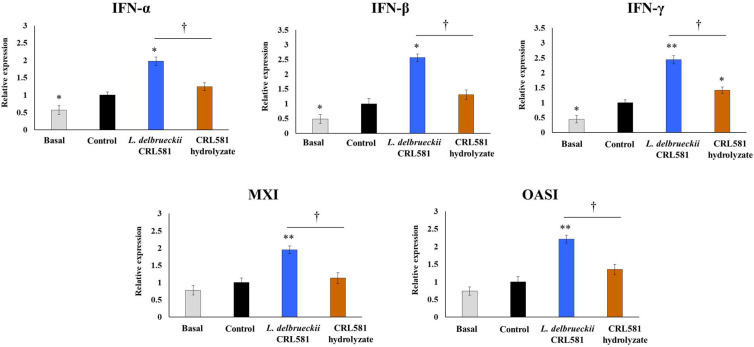
Modulation of the innate antiviral immune response triggered by Toll-like receptor 3 (TLR3) activation in murine macrophages by *Lactobacillus delbrueckii* subsp. *lactis* CRL 581. RAW 264.7 cells were stimulated with *L. delbrueckii* subsp. *lactis* CRL 581 or its β-casein hydrolysate (CRL 581 hydrolysate) and challenged with poly(I:C) to induce the activation of TLR3. RAW 264.7 cells with no treatment (basal) or challenged only with poly(I:C) (control) were used as controls. The expressions of interferon (IFN)-α, IFN-β, IFN-γ, IFN-induced GTP-binding protein Mx1 (Mx1), and 2′-5′-oligoadenylate synthetase 1 (OAS1) were analyzed by qPCR after 12 h of TLR3 activation. The results represent data from three independent experiments. Asterisks indicate significant differences when compared to the control group (* *p* < 0.05, ** *p* < 0.01). Symbols indicate significant differences between the indicated groups (^†^ *p* < 0.05).

We also aimed to evaluate the influence of *L. delbrueckii* subsp. *lactis* CRL 581 on the response of macrophages to the activation of TLR3 signaling pathway. Thus, murine RAW macrophages were stimulated with the CRL 581 strain or the CRL 581 hydrolysate and then challenged with poly(I:C). Twelve hours after TLR3 activation, the expressions of IFN-α, IFN-β, IFN-γ, Mx1, and OAS1 were evaluated (Figure 6).

The challenge of macrophages with poly(I:C) increased the expression of immune factors evaluated in all experimental groups. However, macrophages treated with *L. delbrueckii* subsp. *lactis* CRL 581 had significantly higher levels of IFN-α, IFN-β, IFN-γ, Mx1, and OAS1 than the control groups (Figure 6). The expressions of the antiviral factors in macrophages stimulated with the CRL 581 hydrolysate were not different from controls after poly(I:C) challenge, with the exception of IFN-γ which was increased in the hydrolysate-treated cells (Figure 6). Of note, the expression levels of IFN-γ in hydrolysate-treated cells were statistically different from the observed levels in macrophages stimulated with *L. delbrueckii* subsp. *lactis* CRL 581.

**Figure 7:**
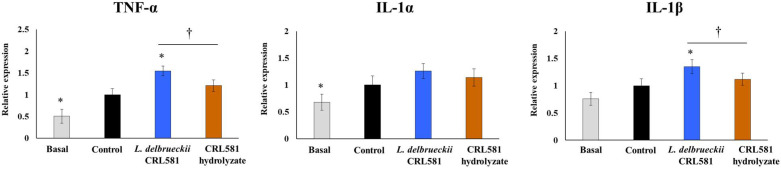
Modulation of the innate antiviral immune response triggered by Toll-like receptor 3 (TLR3) activation in murine macrophages by *Lactobacillus delbrueckii* subsp. *lactis* CRL 581. RAW 264.7 cells were stimulated with *L. delbrueckii* subsp. *lactis* CRL 581 or its β-casein hydrolysate (CRL 581 hydrolysate) and challenged with poly(I:C) to induce the activation of TLR3. RAW 264.7 cells with no treatment (basal) or challenged only with poly(I:C) (control) were used as controls. The expressions of tumor necrosis factor (TNF)-α, interleukin 1α (IL-1α), and IL-1β were analyzed by qPCR after 12 h of TLR3 activation. The results represent data from three independent experiments. Asterisks indicate significant differences when compared to the control group (* *p* < 0.05). Symbols indicate significant differences between the indicated groups (^†^ *p* < 0.05).

TLR3 activation in macrophages also induced an increase in the expression of the inflammatory cytokines TNF-α, IL-1α, and IL-1β (Figure 7). 

Interestingly, macrophages stimulated with *L. delbrueckii* subsp. *lactis* CRL 581 had significantly higher levels of TNF-α and IL-1β than controls, while no effect of this strain was detected for the expression of IL-1α. The expressions of the three inflammatory cytokines in macrophages stimulated with the CRL 581 hydrolysate were not different from controls after the poly(I:C) challenge (Figure 7).

**Figure 8:**
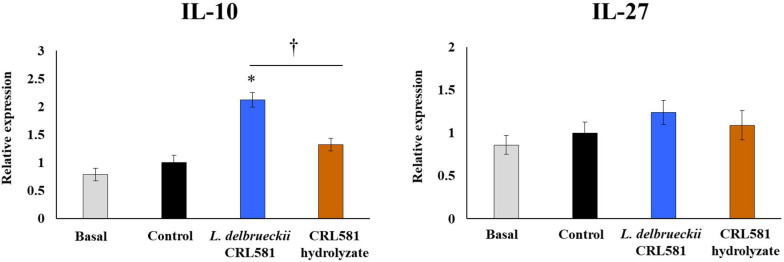
Modulation of the innate antiviral immune response triggered by Toll-like receptor 3 (TLR3) activation in murine macrophages by *Lactobacillus delbrueckii* subsp. *lactis* CRL 581. RAW 264.7 cells were stimulated with *L. delbrueckii* subsp. *lactis* CRL 581 or its β-casein hydrolysate (CRL 581 hydrolysate) and challenged with poly(I:C) to induce the activation of TLR3. RAW 264.7 cells with no treatment (basal) or challenged only with poly(I:C) (control) were used as controls. The expressions of interleukin 10 (IL-10) and IL-27 were analyzed by qPCR after 12 h of TLR3 activation. The results represent data from three independent experiments. Asterisks indicate significant differences when compared to the control group (* *p* < 0.05). Symbols indicate significant differences between the indicated groups (^†^ *p* < 0.05).

Finally, changes in regulatory cytokines produced by macrophages in the context of TLR3 activation were evaluated. As shown in Figure 8, poly(I:C) stimulation did not induce significant changes in the expression of IL-27 at 12 h post-challenge. Neither *L. delbrueckii* subsp. *lactis* CRL 581 nor the CRL 581 hydrolysate were able to induce changes in the expression of IL-27 by macrophages after the activation of TLR3. Of note, the CRL 581 strain significantly augmented the expression of IL-10 in macrophages while this effect was not observed for the CRL 581 hydrolysate (Figure 8).

## 4. Discussion

To the best of our knowledge, only two *L. delbrueckii* spp. strains were shown to improve mucosal antiviral immune responses in vivo. It was demonstrated that *L. delbrueckii* R-1 increased NK cell activity and IFN-γ production by splenocytes after its oral administration to mice [27,28]. Moreover, orally administered *L. delbrueckii* R-1 was capable in improving the resistance of mice against respiratory infection caused by an influenza virus [29]. In addition, we recently showed that *L. delbrueckii* subsp. *lactis* CRL 581 differentially modulated the intestinal antiviral innate immune response in mice after the challenge with the TLR3 agonist poly(I:C) [18]. We hypothesized that the immunomodulatory effects of these *L. delbrueckii* spp. strains could be associated, at least in part, with their ability to improve antiviral responses in IECs due to the interactions of microbe-IECs trigger cellular signaling pathways that modulate effector immune responses [15]. Moreover, the interactions of microorganisms with IECs play an important role in the generation of immune responses since IECs can influence the activation of mucosal immune cells. In this regard, we observed previously that *L. delbrueckii* R-1 increased type I IFNs (IFN-α/β) and the antiviral factors MxA and RNase L expressions after TLR3 signaling pathway activation in PIE cells [30]. In this study, we used the PIE cell in vitro system to analyze the interaction of *L. delbrueckii* spp. *lactis* CRL 581 with IECs and the impact of this interaction with respect to innate antiviral immunity. Our results showed that the CRL 581 strain was able to adhere to PIE cells and differentially regulate the expression of immune factors triggered by TLR3 stimulation.

The capacity of probiotic lactobacilli to adhere to the gut surface is considered a key feature for their interaction with the host and for exerting beneficial effects in the intestinal mucosa. This close microbe–host interaction may provide lactobacilli with the advantage of establishing residence in the intestinal mucosa and may facilitate their contact with IECs and immune cells [31,32]. Adhesion of lactobacilli to mucosal surfaces is a complex process involving several distinct factors from the host and from microorganisms. Surface adhesion molecules of lactobacilli and complementary receptors present on the mucus layer and IECs are key determinants for bacterial adherence. Using porcine systems, we demonstrated here that *L. delbrueckii* spp. *lactis* CRL 581 efficiently adheres to mucins and IECs. Further studies are necessary for determining the set of molecules that allow the CRL 581 strain to possess this property.

**Figure 9:**
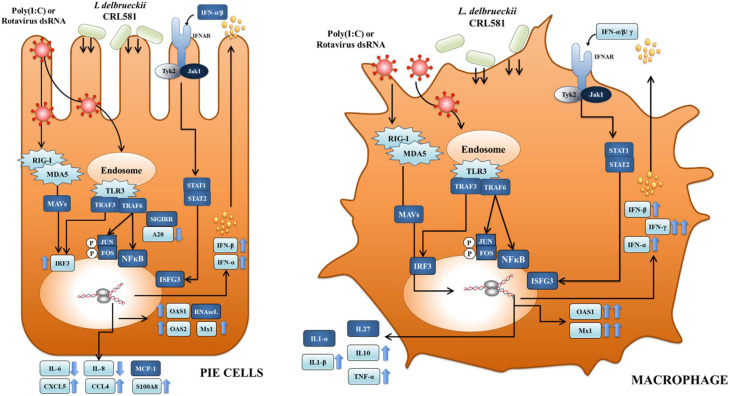
Proposed mechanisms for the modulation of innate antiviral immune responses triggered by Toll-like receptor 3 (TLR3) activation in intestinal epithelial cells and macrophages by *Lactobacillus delbrueckii* subsp. *lactis* CRL 581.

Our previous studies demonstrated that *L. delbrueckii* spp. *lactis* CRL 581 beneficially modulates TLR3-mediated antiviral innate immune response in mice by enhancing the levels of intestinal IFN-β [18]. In line with these previous findings, the transcriptional studies performed here in poly(I:C)-challenged IECs showed that the CRL 581 strain significantly augmented the expression of type I IFNs (IFN-α/β) and the antiviral factors OAS1, OAS2, and MX1. IFN-β expression was stimulated in IECs by rotavirus infection, which then elicited antiviral responses on the same cell and also in surrounding epithelial and immune cells [33,34,35]. IFNs binding to their cell receptors amplifies the expression of IFNs as well as more than 300 different IFN-stimulated genes [36]. Among the hundreds of genes induced by IFN-β in rotavirus-infected IECs, the antiviral factors Mx1 and OAS were shown to be indispensable for viral pathogen clearance. Latent RNAseL is activated by OAS, which in turn restricts rotavirus replication by degrading their RNA molecules. In addition, the RNAseL activity on rotavirus RNA produces small RNA fractions that are recognized by RIG-I amplifying the production of IFN-β [37,38]. Myxovirus resistance proteins (Mx proteins) are intracellular restriction factors that help limit virus replication. Among them, Mx1 was shown to inhibit rotavirus replication by blocking the transcription of viral RNA [39,40]. Of note, we have demonstrated previously that immunobiotic strains able to diminish the increase in A20 in IECs after viral or poly(I:C) challenges could help to enhance the innate immune response mediated by IFNs [22,23,30,41]. A20 is associated to the IKKi/IKKϵ complex suppressing IRF3 dimerization induced by the engagement of TLR3 by viral dsRNA [42]. In this manner, A20 suppresses the IFN-mediated immune responses. Then, immunobiotic bacteria that reduced A20 expression levels in IECs potentiated the IFN-β response [22,43]. Here, we observed a similar effect for *L. delbrueckii* spp. *lactis* CRL 581 in poly(I:C)-challenged PIE cells indicating that this strain may influence IRF3 signaling pathway resulting in an augmentation of IFN production (Figure 9).

We also demonstrated previously that *L. delbrueckii* spp. *lactis* CRL 581 enhances IFN-γ in the intestine of poly(I:C)-challenged mice [18]. Considering that several in vitro and in vivo studies reported that the ability of immunobiotics to enhance IFN-γ is related to their ability to stimulate macrophages [44,45,46], the interaction of the CRL 581 strain with these immune cells was also studied. In our hands, the stimulation of macrophages with *L. delbrueckii* spp. *lactis* CRL 581 significantly improved their ability to express type I IFNs, IFN-γ, antiviral factors, TNF-α, and IL-1β (Figure 9).

Intestinal resident macrophages are the largest population of phagocytes in the body [47]. Conversely, macrophages play an important role in host defense against intestinal infections including those produced by viruses [48,49]. The activation of immune receptors in virus-infected macrophages stimulates the production of IFN-α, IFN-β, IFN-γ, and a variety of inflammatory cytokines that are crucial for innate immunity [48,50]. Macrophages can also secrete inflammatory cytokines via inflammasome activation [51]. The inflammasome complexes induce the maturation of IL-1β and IL-18, which are important in the defense against viral infections because of their ability to regulate leucocyte migration to the infected area as well as to stimulate the activation and polarization of T cells [52]. It was shown that rotavirus is capable in infecting murine macrophages from several tissues, including Peyer’s patches and mesenteric lymph nodes [53,54]. In addition, a significant augment of intestinal macrophages was described in rotavirus-infected gnotobiotic pigs [53,54,55]. Of note, rotavirus-infected macrophage cell lines secrete IFNs and cytokines [56]. In response to rotavirus infection, the RAW264.7 mouse macrophage cell line produces type I IFN, IFN-γ, TNF-α, and inflammatory chemokines [56,57]. Rotavirus replication in bone marrow-derived macrophages also induced IFNs and cytokine production. Thus, macrophages are capable of recognizing rotavirus infection, activating cellular transcription factors, and mediating antiviral cytokine production [8]. IFNs and cytokines secreted by macrophages during rotavirus infection are crucial in controlling virus replication [58,59].

Thus, the improved expression of type I IFNs, TNF-α, IL-1β, and IFN-γ in macrophages treated with *L. delbrueckii* spp. *lactis* CRL 581 indicates that this bacterium is capable of augmenting their antiviral response. In fact, CRL 581-treated macrophages had a significantly higher expression of OAS1 and Mx1. It was shown that the expression of Mx proteins in macrophages is important for antiviral defenses. Elevated levels of IFN are not able to prevent influenza virus replication in mouse macrophages lacking the Mx gene, whereas normal macrophages develop strong antiviral activities mediated by IFN stimulation [60]. OAS1 plays an important role in the resistance to virus mediated by macrophages since polymorphism at the OAS1 locus was implicated in susceptibility to enteroviruses and respiratory syncytial virus [61]. Of note, some studies also demonstrated the ability of immunobiotics to improve resistance to viral infection through modulation of the Mx and OAS expression in macrophages. The treatment of RAW264.7 macrophages with *Lactobacillus gasseri* SBT2055 increases the expression of IFN-β, Mx1, and OAS1. In line with these in vitro findings, it was demonstrated that the oral administration of *L. gasseri* SBT2055 increases the survival rate of mice infected with influenza virus by decreasing its replication in the lung through the enhancement of antiviral factors in macrophages [62]. Our previous in vivo results [18] and the study conducted here indicate that *L. delbrueckii* spp. *lactis* CRL 581 would have great potentials in increasing protection against rotavirus infection. Further in vivo studies are necessary to demonstrate this hypothesis.

The interaction of intestinal-resident macrophages and IECs is an important element of gut innate immunity against pathogens. IECs produce specific cytokines and antimicrobial defense molecules in response to pathogens, which is crucial for activating intestinal mucosal innate and adaptive immune responses [63]. In this regard, it was shown that human IECs produce antiviral factors capable of inhibiting the infection of HIV in macrophages. The study demonstrated that the activation of TLR3 in IECs resulted in the up-regulation of IFN-β, IFN-λ, and CC chemokines. Interestingly, TLR3-activated IECs released exosomes that contained anti-HIV factors, including IFN-stimulated genes and restriction miRNAs. Moreover, macrophages stimulated with supernatant from TLR3-activated IECs cultures were capable of inhibiting the replication of HIV [63]. The detailed study of the indirect modulation of antiviral functions of macrophages by the CRL 581 strain through its action on IECs is an interesting topic for future research, not only for improving the understanding of its benefits in the TLR3-mediated immune response but also for exploring its potential application in other viral infections that target the intestinal mucosa.

Previously, we found that *L. delbrueckii* spp. *lactis* CRL 581 is able to modulate intestinal inflammatory cytokine and chemokine profiles triggered by TLR3 activation in poly(I:C)-challenged mice and is able to protect against mucosal inflammatory damage [18] in a manner similar to that observed for the well-characterized immunobiotic strain *L. rhamnosus* CRL1505 [22,40,64,65]. These results allowed us to speculate that the CRL 581 strain, similar to *L. rhamnosus* CRL1505, would be capable at modulating the expressions of inflammatory factors in IECs. TLR3 pathway has the ability to promote protective immunity as well as inflammatory tissue damage. In this regard, it was shown that IECs produce inflammatory cytokines and chemokines in response to TLR3 activation by rotavirus, including IL-6, IL-8, GM-CSF, and chemokines. As described above, these inflammatory mediators are crucial for the protection against viral pathogens through their direct antiviral effects [66] and the recruitment and activation of immune cells [6]. However, the uncontrolled and excessive infiltration of immune cells to the intestinal mucosa can contribute to local damage [67]. The appropriate regulation of TLR3 pathway is essential for complete protection against rotavirus without detrimental effects. In our hands, *L. delbrueckii* spp. *lactis* CRL 581 diminished the expression of IL-6 and IL-8 in poly(I:C)-challenged PIE cells while the immunobiotic strain augmented the expression of CCL4 and CXCL5 (Figure 9). In addition, excessive and uncontrolled production of TNF-α, IL-1β, IL-6, and other inflammatory cytokines by macrophages may result in serious systemic complications during viral infections such as tissue damage and septic shock [68,69]. Although the treatment of macrophages with *L. delbrueckii* spp. *lactis* CRL 581 augmented the expression of inflammatory factors, enhanced expression levels of the immunoregulatory cytokine IL-10 were also observed (Figure 9). This result is in line with our previous in vivo study in mice in which improved IL-10 levels were observed in the intestinal tract of CRL 581-treated mice after a poly(I:C) challenge [18]. It can be concluded that the differential regulation of inflammatory and anti-inflammatory factors induced by the CRL 581 strain in vivo could be associated with its ability to modulate the responses of both IECs and macrophages.

By using a Crohn’s disease murine model based on the administration of trinitrobenzene sulfonic acid (TNBS)-induction, we demonstrated that CRL 581 β-casein hydrolysate, administered for 10 consecutive days, reduced intestinal inflammatory damage by increasing IL-10 and decreasing IFN-γ production in the gut [70]. However, the administration of these hydrolysates for three consecutive days did not modulate the intestinal immunity triggered by the activation of TLR3 in mice [18]. Similarly, in this study, the modulation of the TLR3-mediated intestinal inflammation in PIE cells and macrophages by CRL 581 β-casein hydrolysate was not observed. These results indicate that CRL 581 β-casein hydrolysate may have anti-inflammatory effects but not the ability to potentiate innate antiviral immune response.

## 5. Conclusions

In conclusion, the results of this study show that the high proteolytic strain *L. delbrueckii* spp. *lactis* CRL 581 was able to beneficially modulate the intestinal innate antiviral immune response by regulating the response of both IECs and macrophages to the stimulation of TLR3. This study also demonstrated that the β-casein hydrolysate is not involved in the ability of the CRL 581 strain to potentiate innate antiviral immune responses. Therefore, this study contributes significantly in improving the understanding of cellular mechanisms involved in the beneficial effect of the CRL 581 strain previously observed in vivo. The in vitro functional studies performed here complemented with our previous in vivo studies in mice demonstrate that *L. delbrueckii* subsp. *lactis* CRL 581 is an interesting immunobiotic strain useful for the development of functional foods with respect to improving protection against viral infections.

## Data Availability

Data is contained within the article.
